# The effects of tea extracts on proinflammatory signaling

**DOI:** 10.1186/1741-7015-4-28

**Published:** 2006-12-01

**Authors:** Frank Pajonk, Anja Riedisser, Michael Henke, William H McBride, Bernd Fiebich

**Affiliations:** 1 Department of Radiation Oncology, David Geffen School of Medicine at UCLA, Los Angeles, CA 90095-1714, USA; 2 Department of Radiation Oncology, University Hospital of Freiburg, Germany; 3 Department of Psychiatry, University Hospital of Freiburg, Germany

## Abstract

**Background:**

Skin toxicity is a common side effect of radiotherapy for solid tumors. Its management can cause treatment gaps and thus can impair cancer treatment. At present, in many countries no standard recommendation for treatment of skin during radiotherapy exists. In this study, we explored the effect of topically-applied tea extracts on the duration of radiation-induced skin toxicity. We investigated the underlying molecular mechanisms and compared effects of tea extracts with the effects of epigallocatechin-gallate, the proposed most-active moiety of green tea.

**Methods:**

Data from 60 patients with cancer of the head and neck or pelvic region topically treated with green or black tea extracts were analyzed retrospectively. Tea extracts were compared for their ability to modulate IL-1β, IL-6, IL-8, TNFα and PGE_2 _release from human monocytes. Effects of tea extracts on 26S proteasome function were assessed. NF-κB activity was monitored by EMSAs. Viability and radiation response of macrophages after exposure to tea extracts was measured by MTT assays.

**Results:**

Tea extracts supported the restitution of skin integrity. Tea extracts inhibited proteasome function and suppressed cytokine release. NF-κB activity was altered by tea extracts in a complex, caspase-dependent manner, which differed from the effects of epigallocatechin-gallate. Additionally, both tea extracts, as well as epigallocatechin-gallate, slightly protected macrophages from ionizing radiation

**Conclusion:**

Tea extracts are an efficient, broadly available treatment option for patients suffering from acute radiation-induced skin toxicity. The molecular mechanisms underlying the beneficial effects are complex, and most likely not exclusively dependent on effects of tea polyphenols such as epigallocatechin-gallate.

## Background

Skin toxicity is a common side effect of external beam radiotherapy in patients suffering from solid cancers. Radiation technique has improved dramatically with the introduction of linear accelerators, thereby reducing skin toxicity. However, current treatment concepts often include radiation dose escalation to improve tumor control, thereby surpassing normal tissue tolerance doses. This often leads to the occurrence of moist skin desquamation (RTOG grade ≥ 2 skin toxicity) [1], which causes discomfort and compromises patients' quality of life during and immediately after treatment. Moreover, clinical management of radiation-induced skin toxicity often requires radiotherapy treatment gaps, thereby reducing the probability of loco-regional tumor control [2]. In the past, many clinical efforts have been made to attenuate skin toxicity in radiation therapy or to delay its onset, but in many countries there is no "gold standard" in skin care during and after radiation treatment. Instead, numerous treatment recommendations exist, which have been established empirically, but many compounds frequently used in these protocols have failed to prove their efficacy in clinical trials [3,4]. To date, the majority of previous studies have aimed to prevent or attenuate skin reactions but did not focus on the duration of radiation-induced skin toxicity, if present.

Patients with grade 2+ skin toxicity mostly suffer from skin disintegrity and symptoms caused by inflammation in the irradiated areas. Radiation-induced inflammation is mainly caused by activation of NF-κB, a preformed dimeric transcription factor sequestered in the cytosol by its inhibitor molecules of the IκB-family. Following ionizing irradiation, IκB-kinases become activated and phosphorylate IκB at specific serine sites. This tags IκB for poly-ubiquitination and subsequent degradation through the 26S proteasome [5], a multi-catalytic, ATP-dependent protease complex, responsible for posttranslational control of all short-lived and many long-lived proteins.

Degradation of IκB frees NF-κB for translocation into the nucleus, where it binds to specific consensus sequences in promoter regions of NF-κB-dependent genes to initiate transcription of pro-inflammatory cytokines [6].

Tea extracts are known to terminate inflammatory conditions, and its usage has been reported to prevent skin damage caused by UV irradiation [7]. There are three types of tea: black, Oolong, and green tea. Green tea is widely consumed in Japan, China, and other Asian nations and is becoming more popular in Western nations. The difference between green tea and the others is that green tea is not fermented, thus preventing antioxidants from being lost during that process. Therefore, and in contrast to black tea, green tea contains high concentrations of polyphenols such as epigallocatechin-gallate (EGCG). Tea polyphenols have been shown to inhibit proteasome function, thereby terminating inflammation [8]. Although tea polyphenols have been claimed to be the most potent constituents of tea, there is increasing evidence that these compounds are not the only constituents responsible for the beneficial effects on heath from tea [9].

In the present study, we analyzed the efficacy of our standard skin care program containing topically-applied black or green tea extracts on the duration of grade 2+ skin toxicity during radiation therapy. We found green tea extracts to be superior to black tea extracts in the treatment of radiation-induced skin toxicity. Investigating the underlying molecular mechanisms to understand how tea extracts help to restore skin integrity, we observed that whole tea extracts altered DNA-binding of the transcription factor NF-κB in a complex manner, different from that seen after EGCG treatment.

## Methods

### Patients

Data from 60 inpatients experiencing skin toxicity RTOG score grade 2 and higher (grade 2+) during radiotherapy at the Department of Radiation Oncology, University Hospital Freiburg, Germany, were assessed retrospectively. All patients received our standard skin care program, which consisted of once-daily treatment of irradiated skin areas with a moisturizing crème (Eucerin Lotion 3% Urea, Beiersdorf AG, Hamburg, Germany) starting at day one of radiation treatment (linear accelerator Clinac, Varian, 6MeV photons; daily fractions of 1.8–2.0 Gy). Frequency of application was increased to twice or three times daily if a radiation-induced erythema occurred, and stopped with the occurrence of moist desquamation. Irradiated skin areas with grade 2+ lesions were treated three times daily with either green or black tea preparations for 10 minutes from the first day moist desquamation was observed. Skin toxicity was scored by nurses on a daily basis before every application of tea extracts. Treatment with tea extracts was discontinued immediately after the disappearance of moist desquamation. Nine patients (four with head and neck cancer, four with cancer in the pelvic region and one patient with gliosarcoma) were released from the hospital with persisting grade 2+ skin toxicity. These patients were censored and the dataset was analyzed for the duration of skin toxicity grade 2+ using the Kaplan-Meier-Method. A p-value ≤ 0.05 (log-rank test) was considered statistically significant. The study was approved by the local ethics board of the University Hospital Freiburg, Germany.

### Cell culture

Cultures of RAW 264.7 murine macrophages (a generous gift of Dr G. Hildebrandt, Department of Radiation Oncology, University of Leipzig, Germany) and MCF-7 breast cancer cells (DMSZ, Braunschweig, Germany) were grown in 75 cm^2 ^flasks (Greiner) at 37°C in a humidified atmosphere at 5% CO_2 _in DMEM medium (Cell Concepts, Umkirch, Germany), supplemented with 10% heat-inactivated FCS and 1% penicillin/streptomycin (Sigma).

### Isolation of human peripheral monocytes

Monocytes from healthy human donors were prepared following a standardised protocol (Ficoll gradient preparation, Amersham-Biosciences) using a completely endotoxin-free cultivation [10,11]. Using 50 ml tubes, 25 ml Ficoll were loaded with 25 ml blood of buffy coats from healthy blood donors. The gradient was established by centrifugation at 1,800 r.p.m., 20°C for 40 min by using slow acceleration and brakes. Peripheral blood mononuclear cells in the interphase were carefully removed and re-suspended in 50 ml pre-warmed phosphate buffered saline (PBS, Invitrogen) followed by centrifugation for 10 min at 1,600 r.p.m. and 20°C. The supernatant was discarded and the pellet washed in 50 ml PBS and centrifuged as described above. The pellet was then re-suspended in 50 ml RPMI-1640 low endotoxin-medium (from Invitrogen) supplemented with 10% human serum (PAA). After counting the amount of cells in a particle counter (Euro Diagnostics, Krefeld), cells were seeded in 24-well plates for ELISA or six-well plates for RNA analysis (1–2 × 10^6 ^cells/well) and incubated at 37°C/5% CO_2_. The medium and the non-adherent cells (lymphocytes) were removed and fresh RPMI-1640 medium containing 1% human serum was added.

### Preparation of tea extracts

Two tea bags (1.5 g each) tea were covered with 50 ml Millipore water (black tea: boiling water; green tea: water at 70°C) for five minutes. The resulting extracts were passed through a 0.8 μM and a 0.22 μM filter to free the extracts from insoluble material. Small aliquots were stored until usage at -20°C in the dark. Given a estimated total catechin content of 10–40 mg/g for green tea [12], tea preparations used in our study, were expected to have an estimated total catechin concentration of 25–100 μg/ml. Epigallocatechin-gallate (Sigma-Aldrich) was solubilized in water and small aliquots of the stock solution were stored until usage at -20°C in the dark.

### Irradiation of cells

Exponentially-growing RAW 264.7 murine macrophages were irradiated at room temperature using a ^137^Cs-laboratory irradiator (IBL 637, CIS bio international) at a dose rate of 0.78 Gy/min. Control cells were sham-irradiated.

### MTT assay

Approximately 10,000 RAW 264.7 murine macrophages per well were plated into 96-well plates in 100 μl RPMI medium per well. After 24 h, tea extracts were added to the media. Three hours later, the cells were irradiated with 0, 2, 4, 6, and 8 Gy. After five days, 20 μl MTT (5 mg/ml) was added to each well and cells were subsequently incubated for four hours at 37°C. Fifty microliters of a 20% SDS solution (0.02 N HCL) was added to each well and absorbance at 560 nm was measured after overnight incubation at 37°C (VersaMax, Molecular Devices).

### Proteasome function assays

Proteasome function was measured as described previously [13] with some minor modifications. To obtain crude cellular extracts, cells were washed with PBS, then with buffer I (50 mM Tris, pH 7.4, 2 mM DTT, 5 mM MgCl_2_, 2 mM ATP), and pelleted by centrifugation (1,000 × ***g***, 5 min, 4°C). Glass beads and homogenization buffer (50 mM Tris, pH 7.4, 1 mM DTT, 5 mM MgCl_2_, 2 mM ATP, 250 mM sucrose) were added and cells were vortexed for 1 min. Beads and cell debris were removed by centrifugation at 1,000 × ***g ***for 5 min and 10,000 × ***g ***for 20 min at 4°C. Protein concentration was determined by the Micro BCA protocol (Pierce) with BSA (Sigma) as standard. To measure 26 S proteasome activity, 10 μg protein of crude cellular extracts or 1 μg protein of partially purified proteasomes of each sample was diluted with buffer I to a final volume of 200 μl. The fluorogenic proteasome substrate SucLLVY-MCA (chymotrypsin-like, Sigma) was dissolved in DMSO and added in a final concentration of 80 μM in 1% DMSO. Proteolytic activity was continuously monitored by measuring the release of the fluorescent group 7-amido-4-methylcoumarin (AMC) in a fluorescence plate reader (SpectraMax Gemini, Molecular Devices, 37°C) at 380/460 nm.

### Cell extracts and electrophoretic mobility shift assays

For preparation of total cytosolic extracts, cells were dislodged mechanically, washed with ice-cold PBS and lysed in TOTEX-buffer (20 mM HEPES, pH 7.9, 0.35 mM NaCl, 20% glycerol, 1% Nonidet P-40, 0.5 mM EDTA, 0.1 mM EGTA, 0.5 mM DTT, PMSF and aprotinin) for 30 minutes on ice. Lysate was centrifuged at 12,000 × ***g ***for 5 min at 4°C. Protein concentration was determined using the BCA protocol (Pierce) and bovine serum albumin (BSA, Sigma) as standard. Fifteen micrograms of protein from the resulting supernatant was incubated for 25 minutes at room temperature with 2 μl BSA (10 μg/μl), 2 μl dIdC (1 μg/μl), 4 μl Ficoll-buffer (20% Ficoll 400, 100 mM HEPES, 300 mM KCl, 10 mM DTT, 0.1 mM PMSF), 2 μl buffer D+ (20 mM HEPES, 20% glycerol, 100 mM KCl, 0.5 mM EDTA, 0.25% NP-40, 2 mM DTT, 0.1 mM PMSF) and 1 μl of the [γ^32^P]-ATP labeled oligonucleotide (Promega, NF-κB: AGT-TGA GGG GAC TTT CCC AGG). For a negative control, unlabeled oligonucleotide was added in 50-fold excess. Gel analysis was carried out in native 4% acrylamide/0.5% TBE gels. Dried gels were placed on a phosphor screen for 24 hours and analyzed on a phosphor imager (Typhoon 9410, Amersham).

### Determination of inflammatory parameters

For the analysis of IL-6, TNF-α, IL-1-β, IL-8 and PGE_2 _release, cells were preincubated with the green and black tea solutions in the indicated concentrations for 30 min before stimulation with LPS (10 ng/ml) for additional 24 h. After culture supernatants were harvested and centrifuged for 10 min at 10,000 × ***g***, levels of the cytokines or PGE_2 _in the supernatant were measured by ELISA (IL-6, IL-8, TNF-α: Pelikine, distributed by HISS, Freiburg, Germany) or EIA (PGE2: AssayDesign, distributed by Biotrend, Köln, Germany) according to the manufacturer's instructions. All experiments were carried out in triplicates using two-sided student's t-test for statistical analysis (a p-value ≤ 0.05 was considered statistically significant).

## Results

### Green tea extracts as supportive care for acute radiation-induced skin reactions

**Figure 1:**
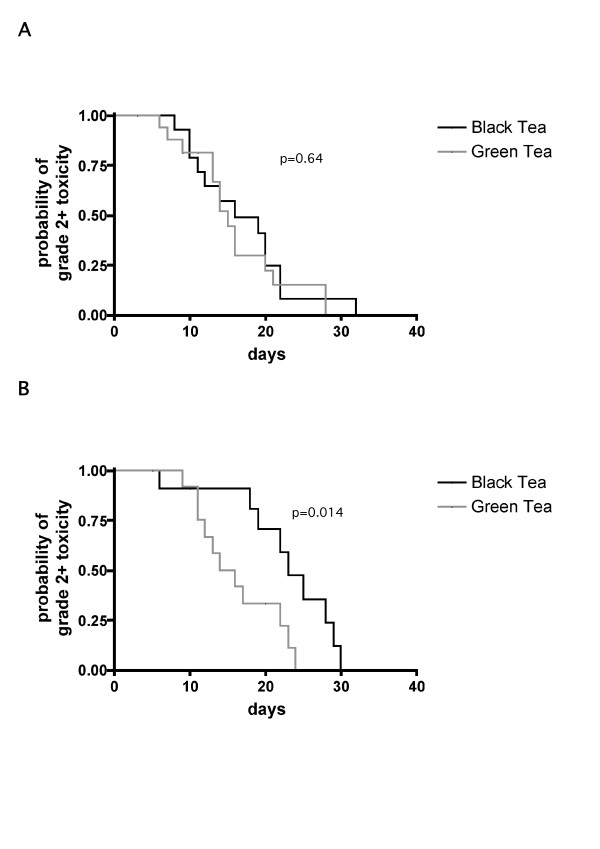
**Duration of radiation-induced grade ≤ 2 skin toxicity in irradiated skin areas**. (A) Patients treated for cancer of the head and neck region. (B) Patients treated for cancer of the pelvic region. p-values: log-rank test (green tea vs. black tea treatment).

During curative-intended radiation treatment of solid cancer, many patients experience grade 2+ skin toxicity, which can cause treatment gaps and thus limit the curability of the cancer. Tea extracts have been applied in our department empirically to terminate acute skin reactions during radiation therapy, but their use has not been investigated systematically to date. In the present study, 60 patients treated with radiation therapy received our standard skin care program. Demographics of all patients are shown in Table 1. The standard skin care program at the Department of Radiation Therapy, University Hospital Freiburg, consisted of thrice-daily topical application of green or black tea extracts to skin regions with grade 2+ toxicity and continued as long as grade 2+ toxicity persisted. Applying these treatments, we found no difference in duration of grade 2+ skin reactions in patients treated for cancer of the head and neck region between green or black tea (Figure 1A, mean duration for green tea 16.5 ± 1.7 days, for black tea 17 ± 1.8 days, p = 0.64; log-rank test). Patients treated for cancer of the pelvic region had a shorter duration of grade 2+ skin toxicity if treated with green tea extracts (Figure 1B, mean duration for green tea 16.3 ± 1.6 days, for black tea 22.2 ± 2.1 days, p = 0.014; log-rank test). The most obvious difference between patients treated for head and neck cancer and patients treated for tumors of the pelvic region was that most of the latter received radiochemotherapy (23/25). Subgroup analysis of patients with cancers of the head and neck region showed no advantage for patients treated with green tea extracts (8/15) during radiochemotherapy. However, the numbers of patients in these subgroups was small and thus the reliability of the statistical analysis was limited.

### Tea extracts inhibit secretion of pro-inflammatory cytokines from activated monocytes

**Figure 2:**
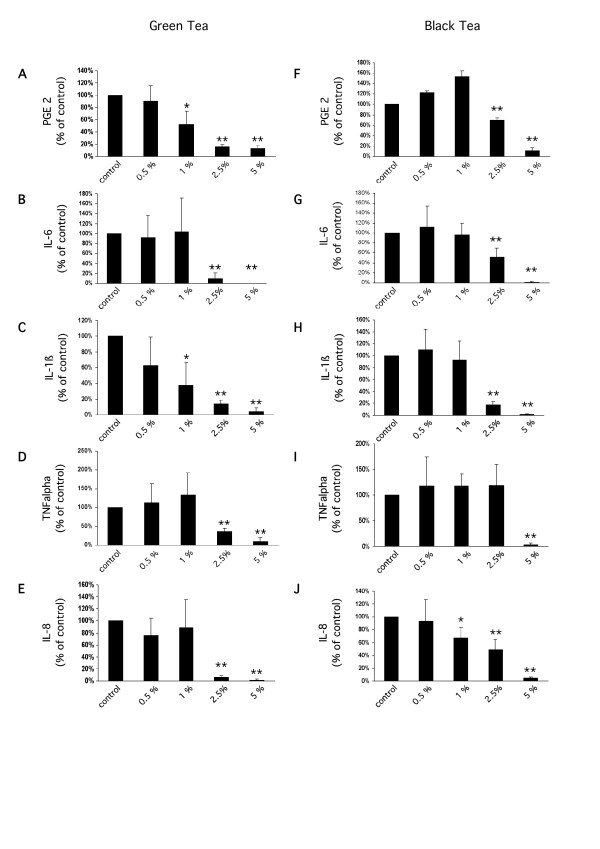
**Release of PGE_2 _(A, F), IL-6 (B, G), IL-1β (C, H), TNFα (D, I), and IL-8 (E, J) from human monocytes after LPS treatment (10 ng/ml) and pre-incubation with green or black tea extracts**. All measurements are expressed relative to the corresponding LPS-treated control (mean ± standard deviation; * p ≤ 0.05, ** p ≤ 0.01, Student's t-test).

Our clinical observations led us to investigate possible underlying molecular mechanisms by which tea extracts could support skin restitution during and after radiotherapy. A hallmark of radiation-induced inflammation is the release of pro-inflammatory cytokines by immune cells. In order to test if clinically-used tea extracts exhibit anti-inflammatory effects in human immune cells we used human monocytes isolated from buffy coats. Both black tea and green tea extracts caused a significant concentration-dependent decrease of LPS-induced IL-1β, IL-6, IL-8, TNFα and PGE_2 _secretion in to the media. While both extracts showed clear anti-inflammatory activity, this inhibitory effect was more pronounced in green tea extracts reflecting the higher efficiency of green tea in the clinical study (Figure 2A–J).

### Clinically used tea extracts have substantial inhibitory effects on proteasome function

To further study the effects of tea extracts on signal transduction in immune cells, we used RAW 264.7 murine macrophages instead of buffy-coat-derived monocytes as a model because it was necessary to scale up the cell number to yield enough protein for proteasome function assays.

Tea extracts contain polyphenols, which have been shown to inhibit proteasome function. Therefore, we decided to test if tea preparations used in skin care during radiation therapy also have inhibitory effects on the proteasome. Crude extracts of exponentially-growing RAW 264.7 macrophages were incubated with serial dilutions of green and black tea extracts. Chymotryptic, tryptic and pepdidyl-glutamyl 26S proteasome activity were continuously monitored using a fluorogenic peptide assay. Green tea extracts caused a concentration-dependent inhibition of the chymotryptic 26S proteasome activity (46% ± 5.3 at 0.5% (p ≤ 0.001; two-sided Student's t-test), 9.9% ± 1.3 at 1% (p ≤ 0.001; two-sided Student's t-test). Green tea extracts at concentrations of 2% and higher completely inhibited chymotryptic 26S proteasome activity.

Extracts from black tea were even more effective. Concentrations of 0.5 % strongly inhibited chymotryptic 26S proteasome activity to 8% ± 1.1 (p ≤ 0.001; two-sided Student's t-test) of untreated controls. No remaining activity was detected at 1% or 0.5%. Comparable results were found for the peptidyl-glutamyl 26S proteasome activity (Table 2), However, inhibition of the tryptic activity by black tea extracts was not dose-dependent at the concentrations used, and was almost already complete at 0.5% concentration.

### Tea extracts cause NF-κB cleavage by a caspase-dependent mechanism

**Figure 3:**
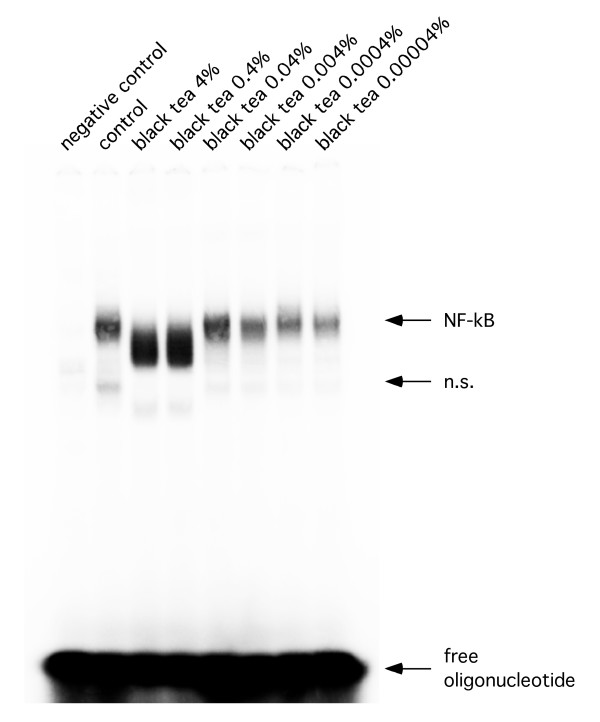
**Representative EMSA**. Representative EMSA using total cellular extracts of RAW 264.7 cells after treatment with black tea extracts.

**Figure 4:**
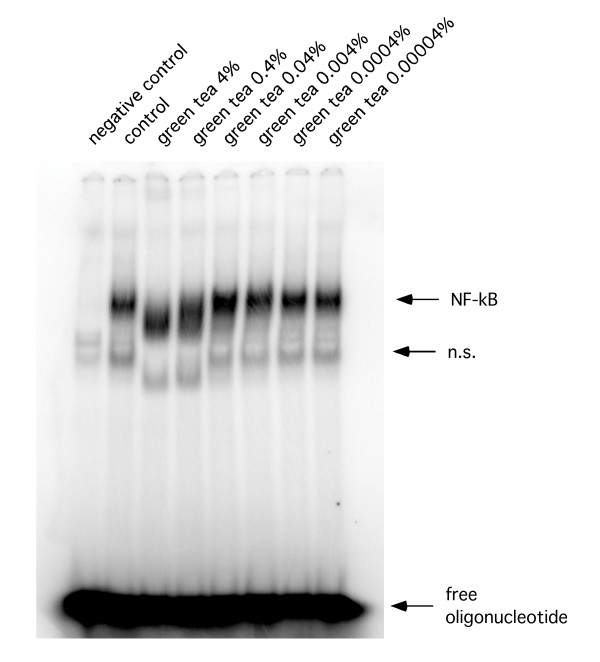
**Representative EMSA**. Representative EMSA using total cellular extracts of RAW 264.7 cells after treatment with green tea extracts.

**Figure 5:**
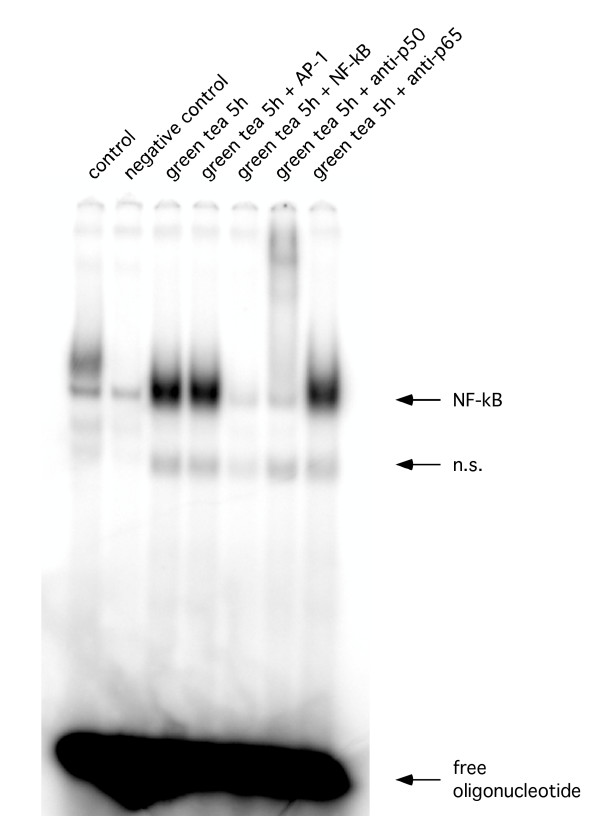
**Representative EMSA**. Supershift assay using an anti-p50 or anti-p65 antibody and lysates from RAW 264.7 cells.

**Figure 6:**
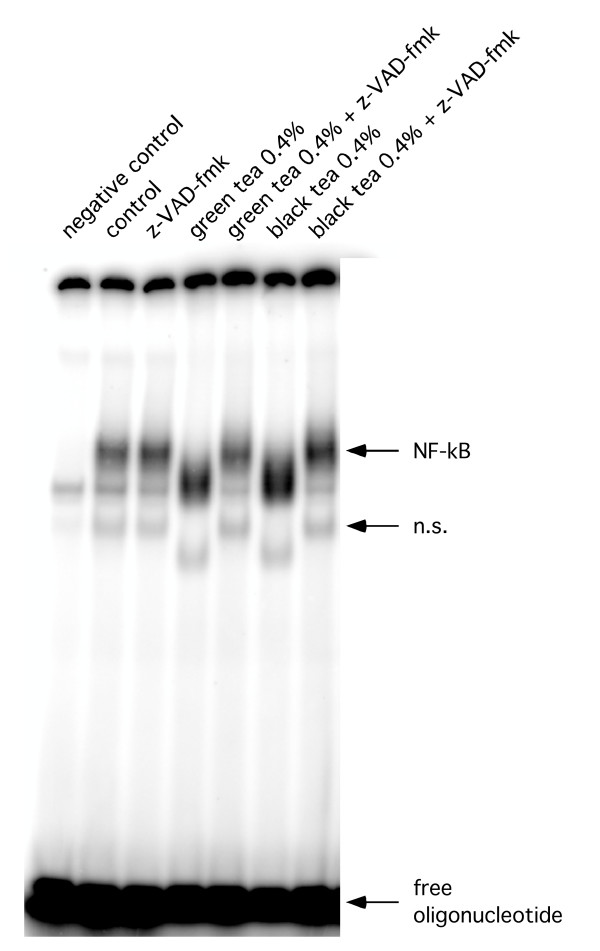
**Representative EMSA**. Representative EMSA using total cellular extracts of RAW 264.7 cells after treatment with black and green extracts and z-VAD-fmk.

**Figure 7:**
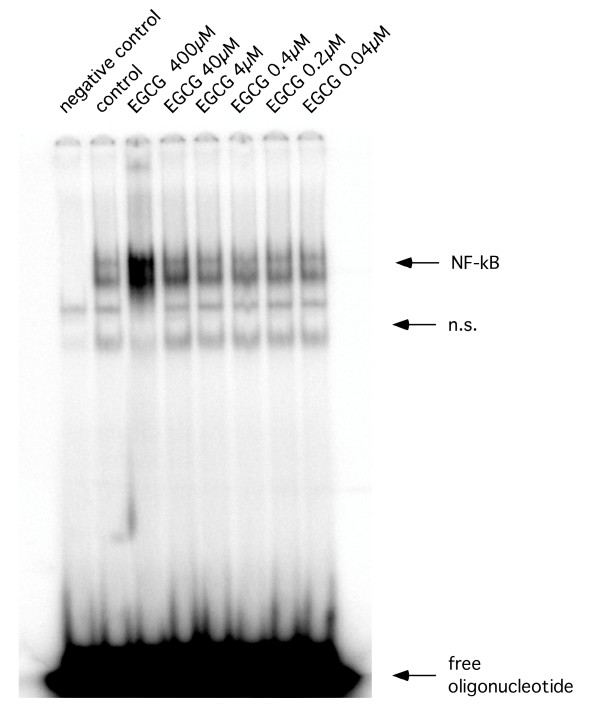
**Representative EMSA**. Representative EMSA using total cellular extracts of RAW 264.7 cells after treatment with black and green extracts and EGCG.

Observing an inhibitory effect of proteasome function, we next tested if – as a consequence – NF-κB DNA-binding activity was affected by tea extracts. Surprisingly, for both extracts we observed an increase in NF-κB activity, which was accompanied by a size -shift of the specific bands at 4% and 0.4% concentrations in the gel-shift assays. Further dilutions caused a decrease in NF-κB DNA-binding activity in experiments using black tea extracts, while the same dilutions of green tea extract had no effect (Figure 3, 4). Supershift assays using an anti-p50 or anti-p65 antibody confirmed specificity of the shifted bands (Figure 5). The observed size shift could be completely reversed by pre-incubation of the cells with the pan-caspase inhibitor z-VAD-fmk (50 μM) indicating involvement of caspases (Figure 6). Further exploring the underlying mechanism, we repeated the gel-shift experiments using EGCG in concentrations comparable to concentrations expected in tea extracts [12]. NF-κB DNA-binding was increased at very high concentrations of EGCG (400 μM), but showed almost no change at all other concentrations used. A size shift of the DNA-bound complex could not be detected (Figure 7).

**Figure 8:**
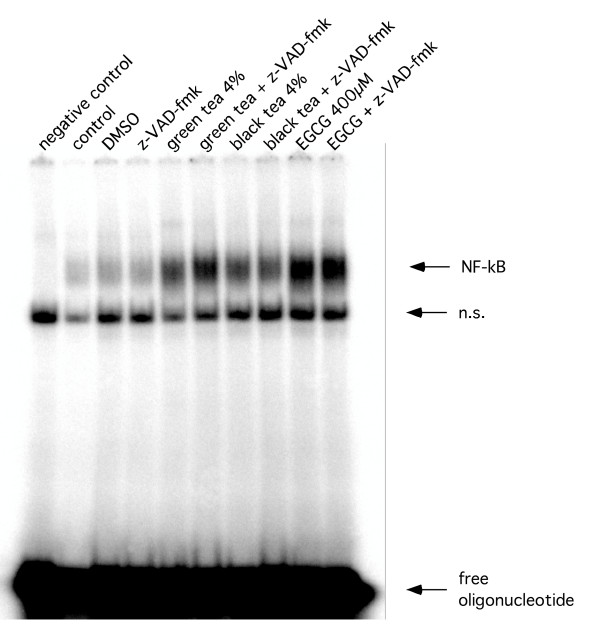
**Representative EMSA**. Representative EMSA using total cellular extracts of MCF-7 cells after treatment with tea extracts, EGCG and z-VAD-fmk.

The pan-caspase inhibitor z-VAD-fmk non-specifically inhibits a broad panel of caspases. In order to further explore the involvement of caspases we used MCF-7 breast cancer cells, which are known to be functionally deleted in caspase-3 [15]. Gel-shift experiments using oligonucleotides containing the consensus binding sequences for NF-κB failed to show a size shift of the specific bands for NF-κB (Figure 8).

**Figure 9:**
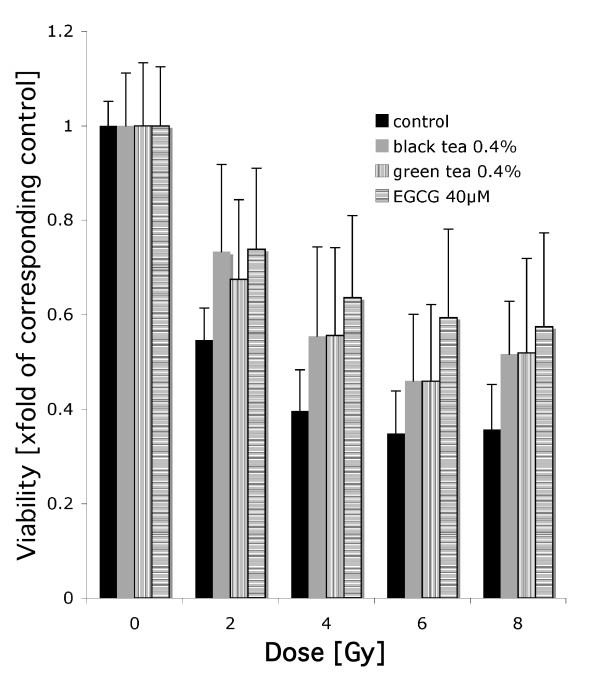
**MTT assay using RAW 264.7 cells after irradiation**. Cells were pretreated with tea extracts or EGCG at indicated concentrations.

We next investigated if tea extracts also altered the viability and radiation response of RAW 264.7 murine macrophages. Cells were pre-incubated with different concentrations of green and black tea extracts for four hours and irradiated with 0, 2, 4, 6 and 8 Gy. EGCG at 400 μM and tea extracts at 4% concentrations caused a significant decrease of viability, consistent with activation of the cellular death program. EGCG at 40 μM or green tea extracts (0.4%) did not have a significant cytotoxic effect while black tea extracts (0.4%) significantly decreased viability to 73.6% ± 8 (p ≤ 0.05; two-sided Student's t-test) of untreated control cells. Concentrations of 0.04% of either green tea or black tea had no toxic effect. Additionally, EGCG (40 μM), green tea extracts (0.4%) and black tea extracts (0.4%) caused a slight radioprotection (Figure 9).

## Discussion

In this study, we showed that a skin care program containing topically-applied tea extracts for radiation-induced grade 2+ skin lesions helped to restore skin integrity. Data on the duration of grade 2+ skin toxicity is very rare in the literature. However, green tea extracts in our study appeared to be an efficient treatment as a previous study reported duration of skin toxicity to be 22 days for anal carcinomas and 26 days for head and neck cancers [16]. Using *in-vitro *models we demonstrated that tea extracts attenuated the release of pro-inflammatory cytokines and terminated pro-inflammatory signaling by caspase-dependent and caspase-independent mechanisms.

In patients with cancer of the pelvic region, in the majority treated with radiochemotherapy, green tea preparations were significantly more effective than extracts from black tea in decreasing the duration of grade 2+ skin toxicity. A possible explanation for the superiority of green tea in patients with cancer of the pelvic region might be the fact that most of these patients suffered from anal carcinomas. By the size, location, and shape of the radiation fields, treatment of anal carcinoma often induces grade 2+ skin toxicity in the inguinal regions and the *rima ani*, and these lesions are prone to bacterial superinfections. Green tea is known for its comparably higher content of polyphenols [12], which exhibit considerable antibacterial activity, especially against gram-positive bacteria such as *S. aureus *[17], often found in superinfected skin lesions of hospitalized patients. The higher content of polyphenols may therefore account for the shorter duration of grade 2+ toxicity in patients treated with green tea extracts. This indicated that the underlying mechanisms of the benefical effects of topically-applied tea extracts are complex and cannot simply be reduced to anti-inflammatory properties of polyphenols.

One of the leading symptoms of radiation-induced dermatitis is inflammation, a major cause of discomfort for patients undergoing radiotherapy. This makes the involved molecular pro-inflammatory pathways a reasonable target for supportive care. Inhibition of the proteasome – a key regulator of inflammation – by tea polyphenols has been reported earlier [8]. In accordance with previous reports, we showed that clinically-used extracts from green and black tea exerted inhibitory effects on all three cleavage activities of the proteasome *in-vitro *and caused a significant decrease in the release of the pro-inflammatory cytokines IL-1β, IL-6, IL-8, TNFα and PGE_2 _in our experimental system.

In addition to their proteasome inhibiting properties, tea polyphenols and theaflavins have been shown to modulate NF-κB activity through the p38 MAPK pathway and direct inhibition of IκB kinases [18,19]. Our results support the existence of an additional method by which tea extracts may modulate the activity of these transcription factors [20]. At high concentrations, tea extracts caused a size shift of NF-κB subunit/DNA complexes. The observation that this size shift occurred less pronounced in MCF-7 cells and that pre-treatment with the pan-caspase inhibitor z-VAD-fmk prevented this effect, indicated involvement of caspases. The increased NF-κB activity at high concentrations of tea extracts may also indicate a possible cleavage of that part of the murine NF-κB subunit p65, which interacts with the inhibitor IκB. Involvement of the N-terminal part of human p65 in IκB binding has been reported [24] but similar studies for murine NF-κB have not been performed yet.

Previously, EGCG has been shown to protect from ionizing radiation, chemotherapy and UV radiation [25-27]. In our study, tea at concentrations causing size shifts of NF-κB complexes reduced the viability of murine macrophages. However, EGCG as well as tea extracts protected the surviving cells from ionizing radiation. EGCG at concentrations similar to those of total tea polyphenols expected in tea extracts [12] did not alter the activity of NF-κB in the same way as whole tea extracts did. This points to a complex mode of action for tea extracts and could indicate that the anti-inflammatory effects of topically-applied tea extracts may at least partially depend on tea constituents different from EGCG.

Taken together, our retrospective analysis of clinical data showed that topically-applied green tea extracts were clinically efficacious in the treatment of grade 2+ skin toxicity during radiation therapy. However, the underlying mechanisms are most likely complex and involve antibacterial and anti-inflammatory processes. Our in-vitro data indicate that these mechanisms involve several different pathways, some of which might be dependent on tea compounds other than polyphenols.

## Conclusion

We conclude that tea extracts are efficient means to treat radiation-induced skin toxicity. Understanding the underlying molecular mechanisms in the future may establish tea extracts as a cheap and broadly available treatment for skin toxicity caused by ionizing radiation in industrial as well as developing countries during clinical radiotherapy and in case of accidents involving ionizing radiation.

## Competing interests

The author(s) declare that they have no competing interests.

## Authors' contributions

FP conceived the study, analyzed the data, and drafted the manuscript. AR conducted the biological studies and participated in drafting the manuscript. MH and WH participated in the design of the study and drafting the manuscript. BF conducted the biological studies and participated in drafting the manuscript.

## Pre-publication history

The pre-publication history for this paper can be accessed here:



## Figures and Tables

**Table 1:** Patient characteristics

	Green tea	Black tea	All patients
Patients (total)	31	29	60
Male:Female	17:14	18:11	35:25
Age (years)	61 ± 2	62 ± 2.1	61 (36–86)
Radiotherapy	11	11	22
Chemoradiotherapy	20	18	38
Total radiation dose (Gy)	57.6 ± 1.9	60.4 ± 2.1	58.9 ± 1 (45–70.4)
Start of tea treatment (Gy)	46.5 ± 1.5	44.4 ± 1.5	45.6 ± 0.8
ite of irradiation			
*Head & neck region*	17	14	31
*Pelvic region*	13	12	25
*Other*	1	3	4
Staging			
*T1*	5	3	8
*T2*	8	10	18
*T3*	10	9	19
*T4*	6	3	9
*Tx*	2	4	6
Leukocytes (Tsd/μl)	5.1 ± 0.2	6.4 ± 0.2	5.7 ± 0.1
Hb (g/dl)	11.3 ± 0.4	11.8 ± 0.4	11.6 ± 0.2
CRP (mg/dl)	3.4 ± 0.1	5.0 ± 0.2	4.2 ± 0.07
BMI (kg/m^2^)	24.5 ± 0.8	24.8 ± 0.9	24.7 ± 0.4

**Table 2:** 26S Proteasome activity

	**Chymotryptic**	**Tryptic**	**Pedidyl-glutamyl**
**Green tea**			
0.5%	46% ± 5.3**	62.7% ± 6.1**	62.7% ± 3.5*
1%	9.9% ± 1.3**	25.7% ± 3.9**	37.1% ± 17.1*
2%	ND	1.8% ± 1.4**	12.6%+ ± 0.7**
**Black tea**			
0.5%	8.0% ± 1.1**	3.1%+ ± 0.7**	50.7% ± 7.1**
1%	ND	10.4% ± 5.7**	11.2% ± 9.3**
2%	ND	6.5% ± 0.001**	ND

*p < 0.05, **p < 0.001, ND: non-detectable

## References

[B1] Cox JD, Stetz J, Pajak TF (1995). Toxicity criteria of the Radiation Therapy Oncology Group (RTOG) and the European Organization for Research and Treatment of Cancer (EORTC). Int J Radiat Oncol Biol Phys.

[B2] Herrmann T, Baumann M (2005). Protraction of waiting time or overall treatment time by unplanned gaps. Clinical importance of compensation [in German]. Strahlenther Onkol.

[B3] Heggie S, Bryant GP, Tripcony L, Keller J, Rose P, Glendenning M, Heath J (2002). A phase III study on the efficacy of topical aloe vera gel on irradiated breast tissue. Cancer Nurs.

[B4] Wells M, Macmillan M, Raab G, MacBride S, Bell N, MacKinnon K, MacDougall H, Samuel L, Munro A (2004). Does aqueous or sucralfate cream affect the severity of erythematous radiation skin reactions? A randomised controlled trial. Radiother Oncol.

[B5] Pajonk F, McBride WH (2001). The proteasome in cancer biology and treatment. Radiat Res.

[B6] Pahl HL (1999). Activators and target genes of Rel/NF-kappaB transcription factors. Oncogene.

[B7] Vayalil PK, Mittal A, Hara Y, Elmets CA, Katiyar SK (2004). Green tea polyphenols prevent ultraviolet light-induced oxidative damage and matrix metalloproteinases expression in mouse skin. J Invest Dermatol.

[B8] Nam S, Smith DM, Dou QP (2001). Ester bond-containing tea polyphenols potently inhibit proteasome activity *in vitro *and *in vivo*. J Biol Chem.

[B9] Widlansky ME, Duffy SJ, Hamburg NM, Gokce N, Warden BA, Wiseman S, Keaney JF, Frei B, Vita JA (2005). Effects of black tea consumption on plasma catechins and markers of oxidative stress and inflammation in patients with coronary artery disease. Free Radic Biol Med.

[B10] English D, Andersen BR (1974). Single-step separation of red blood cells. Granulocytes and mononuclear leukocytes on discontinuous density gradients of Ficoll-Hypaque. J Immunol Methods.

[B11] Noble PB, Cutts JH (1968). Isolation of individual leukocyte types from peripheral blood. J Lab Clin Med.

[B12] Lin YS, Tsai YJ, Tsay JS, Lin JK (2003). Factors affecting the levels of tea polyphenols and caffeine in tea leaves. J Agric Food Chem.

[B13] Glas R, Bogyo M, McMaster JS, Gaczynska M, Ploegh HL (1998). A proteolytic system that compensates for loss of proteasome function. Nature.

[B14] Knuefermann P, Chen P, Misra A, Shi SP, Abdellatif M, Sivasubramanian N (2002). Myotrophin/V-1, a protein up-regulated in the failing human heart and in postnatal cerebellum, converts NFkappa B p50-p65 heterodimers to p50-p50 and p65-p65 homodimers. J Biol Chem.

[B15] Potapova O, Haghighi A, Bost F, Liu C, Birrer MJ, Gjerset R, Mercola D (1997). The Jun kinase/stress-activated protein kinase pathway functions to regulate DNA repair and inhibition of the pathway sensitizes tumor cells to cisplatin. Bio Chem.

[B16] Delaney G, Fisher R, Hook C, Barton M (1997). Sucralfate cream in the management of moist desquamation during radiotherapy. Australas Radiol.

[B17] Taguri T, Tanaka T, Kouno I (2004). Antimicrobial activity of 10 different plant polyphenols against bacteria causing food-borne disease. Biol Pharm Bull.

[B18] Yang F, Oz HS, Barve S, de Villiers WJ, McClain CJ, Varilek GW (2001). The green tea polyphenol (-)-epigallocatechin-3-gallate blocks nuclear factor-kappa B activation by inhibiting I kappa B kinase activity in the intestinal epithelial cell line IEC-6. Mol Pharmacol.

[B19] Pan MH, Lin-Shiau SY, Ho CT, Lin JH, Lin JK (2000). Suppression of lipopolysaccharide-induced nuclear factor-kappaB activity by theaflavin-3,3'-digallate from black tea and other polyphenols through down-regulation of IkappaB kinase activity in macrophages. Biochem Pharmacol.

[B20] Gupta S, Hastak K, Afaq F, Ahmad N, Mukhtar H (2004). Essential role of caspases in epigallocatechin-3-gallate-mediated inhibition of nuclear factor kappa B and induction of apoptosis. Oncogene.

[B21] Ravi R, Bedi A, Fuchs EJ, Bedi A (1998). CD95 (Fas)-induced caspase-mediated proteolysis of NF-kappaB. Cancer Res.

[B22] Thornton MV, Kudo D, Rayman P, Horton C, Molto L, Cathcart MK, Ng C, Paszkiewicz-Kozik E, Bukowski R, Derweesh I (2004). Degradation of NF-kappa B in T cells by gangliosides expressed on renal cell carcinomas. J Immunol.

[B23] Kang KH, Lee KH, Kim MY, Choi KH (2001). Caspase-3-mediated cleavage of the NF-kappa B subunit p65 at the NH2 terminus potentiates naphthoquinone analog-induced apoptosis. J Biol Chem.

[B24] Phelps CB, Sengchanthalangsy LL, Huxford T, Ghosh G (2000). Mechanism of I kappa B alpha binding to NF-kappa B dimers. J Biol Chem.

[B25] Uchida S, Ozaki M, Suzuki K, Shikita M (1992). Radioprotective effects of (-)-epigallocatechin 3-O-gallate (green-tea tannin) in mice. Life Sci.

[B26] Yamamoto T, Staples J, Wataha J, Lewis J, Lockwood P, Schoenlein P, Rao S, Osaki T, Dickinson D, Kamatani T (2004). Protective effects of EGCG on salivary gland cells treated with gamma-radiation or cis-platinum(II)diammine dichloride. Anticancer Res.

[B27] Katiyar SK, Afaq F, Perez A, Mukhtar H (2001). Green tea polyphenol (-)-epigallocatechin-3-gallate treatment of human skin inhibits ultraviolet radiation-induced oxidative stress. Carcinogenesis.

